# Time-resolved cell-to-cell heterogeneity of *Listeria innocua* after nisin exposure

**DOI:** 10.3389/fbioe.2024.1408652

**Published:** 2024-06-12

**Authors:** Niklas Fante, Christian K. Desiderato, Christian U. Riedel, Alexander Grünberger

**Affiliations:** ^1^ Multiscale Bioengineering, Technical Faculty, Bielefeld University, Bielefeld, Germany; ^2^ Department of Biology, University of Ulm, Ulm, Germany; ^3^ Center for Biotechnology (CeBiTec), Bielefeld University, Bielefeld, Germany; ^4^ Institute of Process Engineering in Life Sciences: Microsystems in Bioprocess Engineering, Karlsruhe Institute of Technology, Karlsruhe, Germany

**Keywords:** single-cell analysis, pHluorin2, whole-cell biosensor, bacteriocin, live-cell imaging, microfluidic assay, flow cytometry, persistence

## Abstract

The use of bacteriocins is a promising approach for addressing the immense threat of food-borne and drug-resistant pathogens. In recent years screening platforms for novel bacteriocins using whole-cell biosensors have been established. During screening cell-to-cell heterogeneity is currently neglected but might play a crucial role in signal development of the whole-cell biosensor after bacteriocin exposure. In this study, we explored the temporal dynamics of the signal heterogeneity of the biosensor *Listeria innocua* LMG2785/pNZpHin2^*Lm*^ after nisin exposure using microfluidic single-cell analysis. The results provided novel and detailed insights into the dynamics of cell-to-cell heterogeneity in *L. innocua* LMG2785/pNZpHin2^*Lm*^ at different nisin concentrations with a high spatio-temporal resolution. Furthermore, the formation of subpopulations during bacteriocin exposure was observed. In-depth single-cell tracking even revealed the regeneration of disrupted cells and recovery of pH homeostasis in rare instances. These findings are highly important for the future design and execution of bacteriocin assays and for the interpretation of fluorescence signal development at the population level after exposure to different concentrations of bacteriocins (here, nisin), as well as for obtaining deeper insights into single-cell persistence strategies to quantify the efficacy and efficiency of novel bacteriocins.

## 1 Introduction

Regulatory demands and consumer preferences show an upward trend for minimally processed foods with less chemical additives and preservatives, increasing the demand for innovative natural substitutes with high antimicrobial potency to guarantee food safety (Bangar et al., 2022). At the same time spoilage bacteria and the increasing number of multi-drug resistant pathogens due to decades of antibiotic misuse within agriculture, aquaculture, food industry and the healthcare sector, poses an immense thread for public health (Chao et al., 2021; Pircalabioru et al., 2021; Bangar et al., 2022; Chen et al., 2024). Therefore, there is a high necessity to manage microbial infections, but the development of new antibiotics is not fast enough to cover these needs (Pircalabioru et al., 2021). Novel compounds with antibiotic characteristics, such as bacteriocins, are discussed in this context as possible alternatives to meet the needs in the healthcare and food industry (Pircalabioru et al., 2021; Bangar et al., 2022). They are used as additional safety measures in food processing and have the potential to be applied in future treatment methods in modern medicine (Yang et al., 2014; Lahiri et al., 2022; Verma et al., 2022). For this purpose, insights into the antibiotic potential of novel bacteriocins are necessary to assess their efficacy and determine whether they fulfill the relevant requirements and expectations. Potentially suitable substances can be tested with novel assay approaches using whole-cell biosensors that can react directly to bacteriocins and show quantifiable effects on a given pathogenic organism. An approach using whole-cell *Listeria* biosensor strains has already shown great potential for testing novel compounds (Desiderato et al., 2021; Reich et al., 2022). The pore-forming and lipid II-aggregating lantibiotic nisin (Brötz et al., 1998; Scherer et al., 2015), which has shown potential as a food and feed additive (Younes et al., 2017; Ibarra-Sánchez et al., 2020; Kierończyk et al., 2020) as well as in medical applications (Shin et al., 2016; O'Reilly et al., 2023; Chan et al., 2023), is the only bacteriocin that is approved by the FDA and EFSA as a biopreservative and food additive (Younes et al., 2017; Chan et al., 2023). Thus, nisin is an ideal standard bacteriocin for the evaluation of whole-cell biosensors and for comparison with other methods and sample types, e.g., culture supernatants of natural producers. The whole-cell *Listeria* biosensor mentioned above has a fluorescence readout that is coupled to the internal pH of the sensor cells and measures the impairment of pH homeostasis caused by membrane damage after nisin exposure. A recent study (Reich et al., 2022) investigated the signal output of the sensor strain, but the heterogeneity of the sensor output was not further investigated. However, *L. monocytogenes* showed heterogeneous behavior at the single-cell level after nisin treatment (Budde and Jakobsen, 2000). Therefore, cell-to-cell heterogeneity might play a major role in the formation of intermediate signals. Especially at lethal concentrations cell-to-cell heterogeneity could indicate persistence strategies with indications for both the sensor robustness for quantification and the susceptibility of the respective pathogens against bacteriocins. Accordingly, a better understanding and assessment of the reliability of whole-cell biosensors in light of the development of cell-to-cell signal heterogeneity when exposed to cytotoxic substances is needed.

In this study, we investigated the temporally resolved dynamics of the development of cell-to-cell heterogeneity in the ratiometric, pH-dependent pHluorin2 fluorescence signal of the *L. innocua* LMG2785/pNZpHin2^*Lm*^ whole-cell biosensor after exposure to membrane-damaging nisin. We used a microfluidic single-cell cultivation setup in combination with fluorescence microscopy to show the development of nisin concentration-dependent cell-to-cell signal heterogeneity in this *L. innocua* whole-cell biosensor strain. The results provide a detailed understanding of how nisin affects cells and are important for the application and interpretation of state-of-the-art inhibition assays, e.g., microtiter plate (MTP) assays, which often lack single-cell data.

## 2 Materials and methods

### 2.1 Bacterial strain and sensor principle

A biosensor derived from *Listeria innocua* LMG2785 was used in this study. This bacterial strain serves as a nonpathogenic surrogate organism for *Listeria monocytogenes*, a major foodborne human pathogen (Hoffmann et al., 2012; Sarno et al., 2021). *Listeria innocua* is a Gram-positive bacterium that is susceptible to various bacteriocins, including nisin. The biosensor strain contains the pNZ-pHin2^*Lm*^ plasmid, which encodes the pHluorin2 gene under the control of the constitutive P_help_ promoter. Plasmid stability is ensured by chloramphenicol (10 μg mL^−1^) resistance (Reich et al., 2022).

The pHluorin2 fluorophore has two pH-dependent excitation maxima at approximately 400 and 480 nm (Mahon, 2011; Reich et al., 2022). After excitation at these peaks, the fluorescence intensity at 520 nm changes in a ratiometric, pH-dependent manner. Thus, the extent of the effects of nisin on individual cells after pore formation in the cell wall and the disruption of pH homeostasis in a slightly acidic environment can be measured (Crauwels et al., 2018). The relative fluorescence units (RFU, emission at 520 nm) after excitation with the two excitation maxima were measured to determine changes in the intracellular pH by calculating the ratio of the two values according to the following formula:

$$\text{ratio }{\text{RFU}}_{400/480}=\frac{{\text{RFU}}_{400}}{{\text{RFU}}_{480}}$$

### 2.2 Media and chemicals

The complex medium brain-heart-infusion (BHI) (Carl Roth GmbH + Co. KG, Germany) is commonly used for cultivating *Listeria* species (Jones and D'Orazio, 2013) and was used for precultivation of the biosensor strain. The pH was adjusted to 7.4 with hydrochloric acid or sodium hydroxide if needed, and the BHI base solution was autoclaved. Shortly before inoculation, sterile-filtered chloramphenicol from stock solution was added to the medium to a final concentration of 10 μg mL^−1^. A chloramphenicol stock solution with a concentration of 34 mg mL^−1^ was prepared by diluting chloramphenicol sulfate in 100% ethanol.

The optimized *Listeria* minimal buffer (LMBO: 200 mM MES, 4.82 mM K_2_HPO_4_, 11.55 mM Na_2_HPO_4_, 1.7 mM MgSO_4_, 0.6 mg mL^−1^ (NH_4_)_2_SO_4_, and 55 mM glucose) differed slightly in the buffer used (MES instead of MOPS) and the pH (6.2 instead of 6.5) from the previously published LMB (Crauwels et al., 2018), which was based on modified synthetic minimal medium for *L. monocytogenes* (Tsai and Hodgson, 2003). It was prepared in distilled H_2_O, and the pH was adjusted to 6.2 with hydrochloric acid or sodium hydroxide. Afterward, the solution was sterile-filtered and stored at 4 °C. Shortly before inoculation, sterile-filtered chloramphenicol stock solution was added to the buffer to a final concentration of 10 μg mL^−1^.

A 250 μg mL^−1^ nisin stock solution was prepared by diluting commercial nisin (2.5% nisin balance sodium chloride, Merck KGaA, Germany) in distilled H_2_O. After mixing and sterile filtration, the solution was divided into aliquots and stored at −20 °C.

### 2.3 Precultivation procedure

*Listeria innocua* LMG2785/pNZ-pHin2^*Lm*^ was precultivated in 100 mL baffled shaking flasks in 10 mL of BHI medium supplemented with 10 μg mL^−1^ chloramphenicol. The culture was inoculated with 200 µL of inoculum taken from a glycerol stock and cultivated overnight at 37 °C on a shaker at 120 rpm.

### 2.4 Microtiter plate-based analysis

**FIGURE 1:**
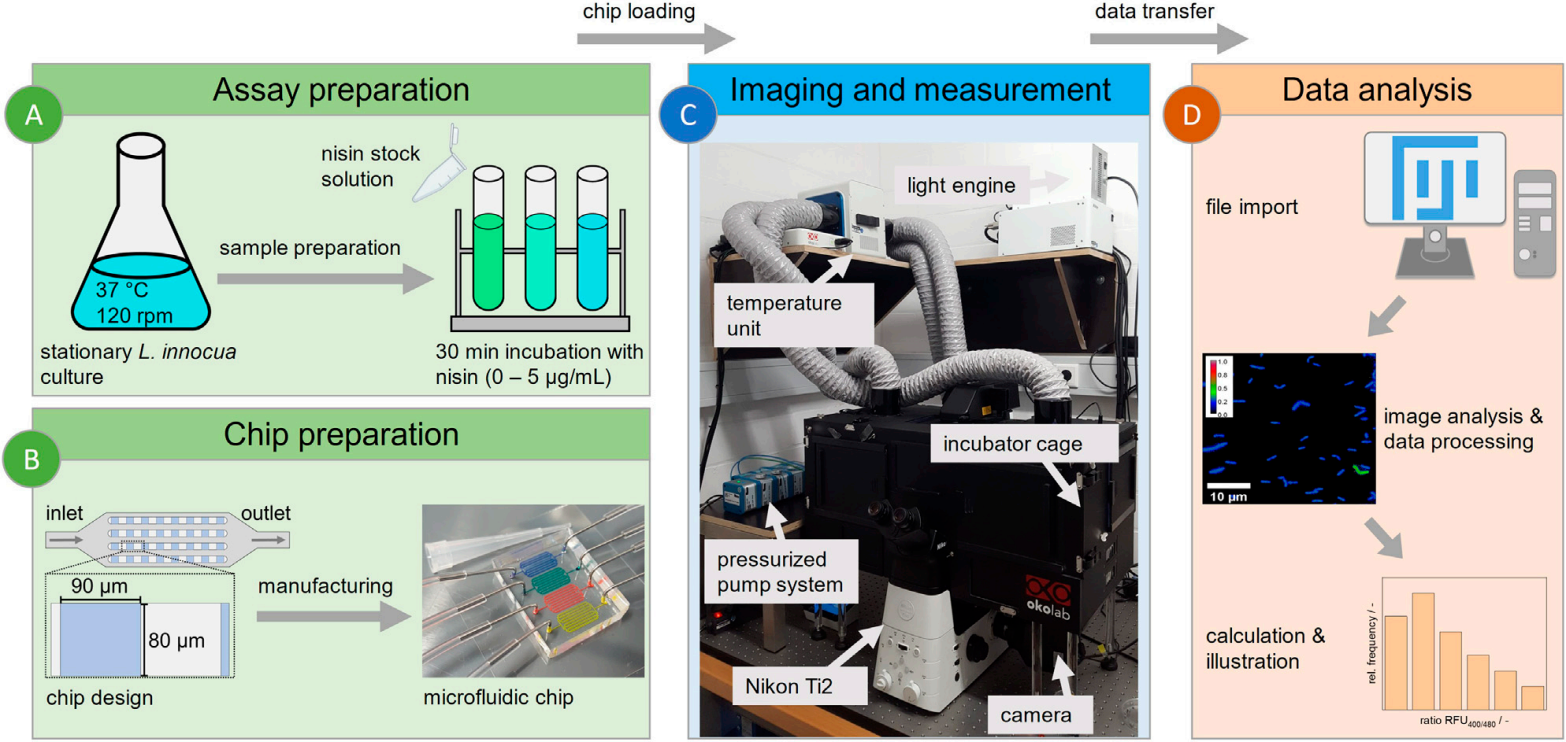
Workflow for microfluidic single-cell analysis. **(A)** Cultivation of *L. innocua* biosensor and sample preparation. **(B)** Design and manufacture of a PDMS-based microfluidic chip with 90 × 80 µm monolayer analysis chambers. **(C)** Technical setup of experiment for imaging, featuring the following peripherals: a Nikon Ti2 fluorescence microscope with an incubator cage, a pressurized pump system and a medium reservoir and waste. **(D)** Workflow of image-based data analysis using Fiji for semiautomated data acquisition and evaluation.

An overnight culture of *L. innocua* LMG2785/pNZ-pHin2^*Lm*^ was washed once with phosphate-buffered saline (PBS) and diluted in LMBO buffer to an OD_600_ of 3. Then, 100 µL of cell suspension was mixed with 100 µL of nisin solution in a black microtiter plate. The nisin concentration used in the pHluorin2 assay ranged from 0 to 5 µg mL^−1^ nisin. After the addition of nisin, the assay mixture was incubated for 30 min in the dark at room temperature (Figure 1A). The emission of pHluorin2 at 520 nm (excitation at 400 and 480 nm) was recorded with a multimode plate reader (Infinite^®^ M200, Tecan, Switzerland).

### 2.5 Flow cytometry-based single-cell analysis

A flow cytometer (FC) (Amnis^®^ CellStream^®^, Luminex, United States) was used to analyze nisin-treated *L. innocua* LMG2785/pNZ-pHin2^*Lm*^ as described previously (Reich et al., 2022). Biosensor cells were diluted 1:50 in PBS, and 50 µL of cell suspension was analyzed for pHluorin2 fluorescence emission at 528 nm using 405 and 488 nm laser irradiation. The laser powers used were 10% (forward scatter and side scatter), 35% (405 nm) and 40% (488 nm). The flow speed was set to “slow” for maximal sensitivity. Gating was performed as described by Reich et al., 2022. The flow cytometry data were analyzed using FlowJo (v10) software.

### 2.6 Chip design and fabrication

A polydimethylsiloxane (PDMS)-based microfluidic system was used for cell trapping and time lapse imaging (Grünberger et al., 2015). The chip design features inlet and outlet structures for loading cells and injecting nisin to maintain a stable nisin concentration and defined conditions during measurement. Cell trapping was achieved by several chambers (80 × 90 µm) with a height of approximately 650 nm arranged in multiple arrays, as shown in Figure 1B. A silicon wafer containing the negative structure was used as a mold for fabrication. Two-component PDMS was mixed at a base:linker ratio of 10:1 (Sylgard 184 Silicone Elastomer, Dow Corning Corporation, United States) and used to cast the structure of the wafer. After the air was removed in a desiccator and the mold was baked at 80 °C for 2 h, the mold was cut out and prepared for plasma bonding. This included cleaning the glass substrate (D 263 T eco, 39.5 × 34.5 × 0.175 mm, Schott, Germany) and the mold itself with analytical-grade isopropanol as well as punching the inlet and outlet structure with a 0.75 mm biopsy puncher (Reusable Biopsy Punch, 0.75 mm, WPI, United States). After surface activation in a plasma generator (Diener Femto Model 1 B2, Diener electronic, Germany), the PDMS mold was placed on a glass substrate for covalent bonding, creating closed structures and a functional microfluidic chip. For a detailed fabrication protocol the reader is referred to Gruenberger et al. (Gruenberger et al., 2013).

### 2.7 Microfluidic setup and live cell imaging

Time lapse imaging was performed with an inverse microscope (Nikon Eclipse Ti2 Series, Nikon, Germany) (Figure 1C). After fabrication, the microfluidic chip was fixed on an in-house manufactured sample plate and loaded with stationary cells from the preculture as described above (Figure 1A).

Data from the MTP assay heterogeneity comparison experiments (chapter 3.1) were collected using a sample from the preculture that was prepared by a procedure similar to that described for the microtiter plate assay. The sample was loaded on the chip shortly before the end of nisin incubation and measured after 30 min of nisin application. For temporally resolved experimental data (chapters 3.2 and 3.3), the loaded sample was obtained immediately after mixing the stationary culture with nisin. During the measurement, the chip was perfused with media with the tested nisin concentrations using a pressurized pump system (Microfluidic Flow Control System™, Fluigent, Germany) to eliminate changes in concentration due to solvent evaporation or nisin degradation. After inoculation of the microfluidic chip, the chamber area was homogenously covered with scattered single cells to counteract limitation-like effects and decrease the possibility of nisin deficiency in supply channels to a negligible level. Furthermore, cell-to-cell fluorescence heterogeneity effects can be distinguished from effects that can be attributed to cell proliferation.

Phase contrast microscopy was used to identify chambers suitable for measurement; the region of interest (ROI) was marked, and the possible movement of cells over time was checked to exclude false values due to deviations in the cell position and chosen ROI. Quantitative fluorescence signal measurements were conducted using two different fluorescence filter combinations with the same emission filter to represent the two pHluorin2 excitation maxima at 400 nm and 480 nm. The illumination intensity was regulated using a light engine (SOLA Light Engine, Lumencor, United States) and was set to 10% to reduce photobleaching.

### 2.8 Image and data analysis

The standard workflow for data analysis is shown in Figure 1D. Image processing and analysis were performed using the Fiji distribution of the open-source software ImageJ (Schindelin et al., 2012). The cells and regions for the endpoint measurements 30 min after nisin exposure (Figure 3) were selected and measured manually, whereas images for dynamic heterogeneity analysis (Figure 6; Figure 7) were obtained with automatic built-in tools in Fiji. As a first step, the cells were identified in the phase contrast image using the “Analyze Particles” feature, and watershed transformation was applied to separate multiple single cells that were initially identified as one cell. Fine-tuning of selected regions was carried out manually. Image stabilization was performed prior to selecting the regions, if necessary. After checking and applying the ROI for every timepoint and fluorescence channel, the fluorescence values were measured as the grayscale value of the light intensity at the image sensor. The ratiometric signal of each cell at each timepoint was then calculated from the maximum values of the respective cell in both fluorescence channels after subtracting the mean background signal value from the fluorescence value. While maximum values generally might not be as robust as the mean value for the assignment of a determined value to a specific cell, the negative impact of manual analysis and differing parameters for ROI identification on the obtained data is greater for the mean, which is thus more prone to error. The determination of the blank value of each image was the opposite. Single pixels could be overexposed due to impurities, refraction or reflection phenomena, leading to high impacts on the determined signal when calculating fluorescence ratios. The mean values over an extended area of the chamber were more reliable and descriptive of the background signal. Cells that did not show a notable signal higher than the background were not taken into account and were excluded from the measurement to prevent false distortion of the measuring principle and signal heterogeneity.

Signal heterogeneity can be quantified and displayed as the standard deviation of all measured single-cell values, whereas the mean value of all measured cells corresponds to the mean sensor signal of the population at the respective nisin concentration. This statistical evaluation method also addresses outlier values, which must be considered when analyzing signal heterogeneity at the single-cell level, as the signals of individual cells can differ immensely from the mean value of a given population.

Similar to the gating method used in flow cytometry analysis (see Supplementary Material, Supplementary Figure S1), the cutoff of the distributions can be used to divide the cultures into two populations and to define signal ranges in which the cells can be classified as “dead” or “alive”. In the microfluidic setup, the signal range for living cells was greater than approximately 0.5 (Figure 3, 0 μg mL^−1^ nisin), and the signal range for dead cells was a ratiometric fluorescence signal less than approximately 0.4 (Figure 3, 5 μg mL^−1^ nisin).

## 3 Results and discussion

Cell-to-cell heterogeneity is an important characteristic that must be considered when characterizing whole-cell biosensors. Thus, novel technologies for the investigation of possible cell-to-cell heterogeneity in sensor strains need to be established and utilized. Here, a microfluidic setup was used in addition to microtiter and flow cytometry-based analyses to acquire insights into the time-dependent development of cell-to-cell signal heterogeneity at the single-cell level.

### 3.1 Nisin sensitivity on different systems

#### 3.1.1 Microtiter plate-based analysis

In the first set of experiments, the susceptibility of stationary *L. innocua* LMG2785/pNZ-pHin2^*Lm*^ cells to different concentrations of the lantibiotic nisin (up to 5 μg mL^−1^) was investigated using a microtiter plate assay for bulk-scale quantification of nisin efficacy.

**FIGURE 2:**
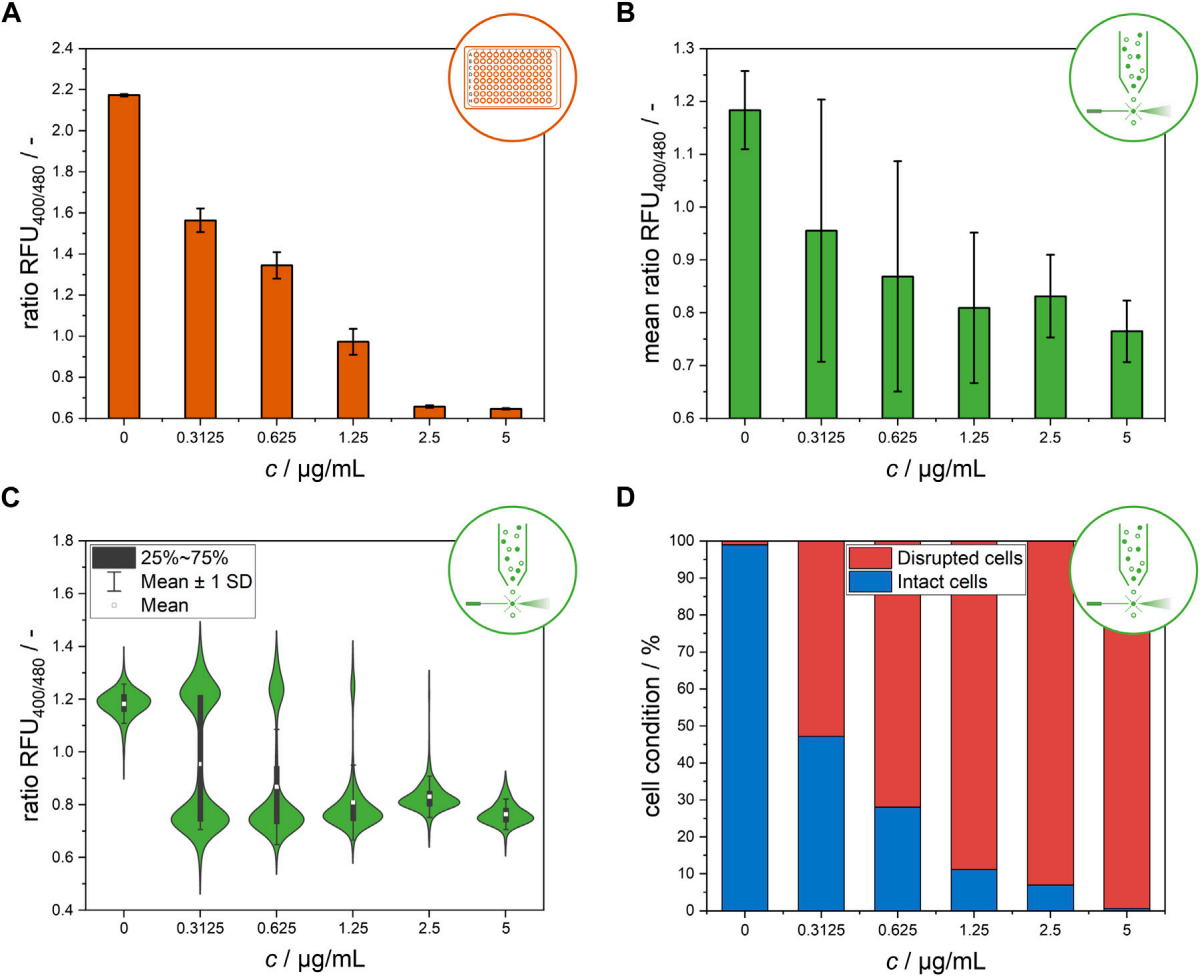
Endpoint measurement of the ratiometric fluorescence signal of the *L. innocua* biosensor after 30 min of nisin exposure at different concentrations. **(A)** Bulk-scale microtiter plate-based results, presented as the mean values of the ratio RFU with respective standard deviations of triplicate measurements. **(B)** Flow cytometry-based results at single-cell level, presented as the mean values of the ratio RFU of all single cells and the respective standard deviations. **(C)** Distribution of single-cell ratio RFU values obtained from flow cytometry measurement. **(D)** Flow cytometry-based results after classification of single-cell signals and subdivision into disrupted and intact cells.

When the effect of nisin exposure on the *L. innocua* biosensor strain across the entire population was analyzed via MTP assays, a clear trend regarding the ratiometric relative fluorescence (ratio RFU) was observed. Exposure to increasing nisin concentrations resulted in a successive decrease in ratio RFU values (Figure 2A). Notably, the standard deviations of the ratio RFU values were 10–17 times greater at intermediate concentrations of 0.3125, 0.625 and 1.25 μg mL^−1^ nisin than at 0 or 5 μg mL^−1^ nisin. These high standard deviations might be caused by underlying cell-to-cell heterogeneity in the susceptibility of the biosensor strain population to different nisin concentrations.

#### 3.1.2 Flow cytometry-based single-cell analysis

To address potential cell-to-cell heterogeneity, stationary *L. innocua* LMG2785/pNZ-pHin2^*Lm*^ cells were investigated at the single-cell level using flow cytometry analysis (Figures 2B–D).

Figure 2B shows a clear trend of a decreasing ratio RFU with increasing nisin concentration. Similar to the data from the MTP assays, standard deviations at intermediate nisin concentrations were dramatically higher than those at high and low nisin concentrations, indicating high cell-to-cell heterogeneity within these populations (Figure 2B). In contrast, cells within populations that were exposed to very high levels of cytotoxic stress at 5 μg mL^−1^ nisin or no stress at all in the 0 μg mL^−1^ nisin control group behaved quite uniformly. These results suggest that, especially at intermediate concentrations, cell-to-cell heterogeneity may play an important role in the formation of the mean signals. This information is critical for application and concentration determination since nonuniform behavior has a high impact on measured signals and makes it considerably more difficult to distinguish between heterogeneity and measurement error. Therefore, determining concentration from the quantification of the sole ratiometric fluorescence signal based on the mean value of all single cells is difficult because the mean values of all measured cells are not stable and distinguishable from one another at all intermediate concentrations tested (Figure 2B). To better understand these high standard deviations at intermediate nisin concentrations, the ratio RFU values were analyzed at the single-cell level (Figure 2C). This analysis revealed two distinct subpopulations at nisin concentrations of 0.3125–1.25 μg mL^−1^. At these concentrations, the cells were clearly separated into two subpopulations with ratio RFU values corresponding to disrupted and nondisrupted cells. As expected, the subpopulations of intact and disrupted cells shifted toward disrupted cells as the nisin concentration increased, confirming the assumption of the relevance of heterogeneity for previous measurements in microtiter plates or flow cytometers. The results are consistent with a previous study that showed the development of two subpopulations of *L. monocytogenes* at subinhibitory concentrations of leucocin 4,014 and nisin, showing the bacteriocin concentration dependent susceptibility of individual cells rather than the whole population (Hornbaek et al., 2006). The formation of heterogeneity in the form of heteroresistance was observed in similar studies. Clonal heteroresistance was detected in an isogenic bacterial population of *Escherichia coli* after treatment with the antibiotic cefotaxime (Scheler et al., 2020) and heterogeneity profiles of biofilms from *Staphylococcus aureus* indicating the triggering of heteroresistance after gentamicin exposure at sub-minimal inhibitory concentrations (Pacocha et al., 2022).

The clear distribution enables the discrimination of single-cell signals belonging to intact and disrupted cells by gating the flow cytometry data (Supplementary Material, Supplementary Figure S1) (Reich et al., 2022). The subpopulation classification results are shown in Figure 2D as percentage values of the cell conditions. In contrast to the use of the mean signal to determine nisin efficacy (Figure 2B), the classification of cells into two subpopulations according to their single-cell signals reveals a clear distinction between samples treated with different nisin concentrations and demonstrates a feasible method for the determination of nisin concentrations using flow cytometry. This finding shows that the results from flow cytometry and microtiter plate-based setups can still be compared if the right evaluation method is chosen. Furthermore, these single-cell data revealed insights into the distribution of ratiometric fluorescence signals and therefore signal heterogeneity within the *L. innocua* biosensor population.

#### 3.1.3 Microfluidics-based signal quantification at single-cell level

**FIGURE 3:**
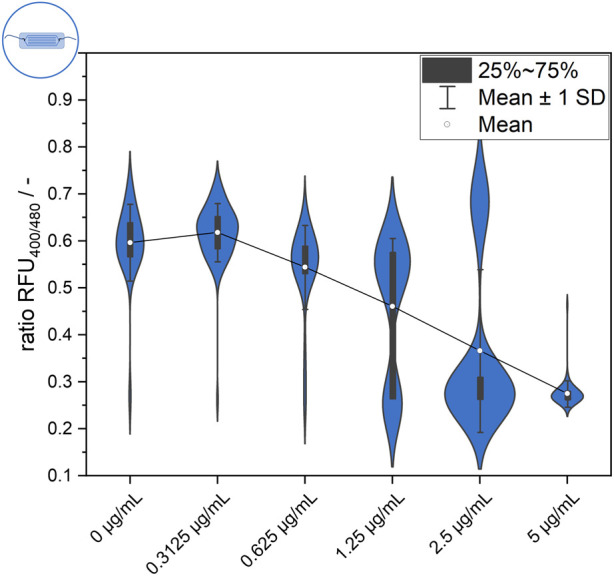
Endpoint measurement of ratiometric fluorescence signal of the *L. innocua* biosensor in the microfluidic setup after 30 min of exposure to different nisin concentrations.

Single-cell behavior was further investigated using a microfluidic setup for single-cell trapping and analysis. Data obtained from single-cell analysis enable comparison between systems and comparison of data from endpoint measurements after 30 min to the time-resolved dynamic signal development measurements afterward. Figure 3 shows the distribution of single-cell values after 30 min of incubation at each nisin concentration. Two distinctive subpopulations appear at a ratiometric fluorescence value of approximately 0.6, which is considered an “alive” signal, and at approximately 0.25, which is considered a “dead” signal (see Materials and Methods). In general, a steadily increasing proportion of dead cells can be observed with increasing nisin concentration. If the cells are separated into two main populations, cell-to-cell heterogeneity within the subpopulations can also be observed. For example, the signal of live cells in the negative control (0 μg mL^−1^ nisin) ranged from approximately 0.5–0.75. The same applies to high cytotoxic stress at 5 μg mL^−1^ nisin, where the ratiometric fluorescence signal for “dead” cells ranges from approximately 0.25–0.3. However, it is also evident that in the negative control (0 μg mL^−1^ nisin) as well as at high nisin concentrations (5 μg mL^−1^ nisin), individual cells can deviate from the main population. For example, even in the absence of cytotoxic stress, some cells cannot maintain their pH equilibrium at 0 μg mL^−1^ nisin and exhibit a dead signal. A few cells that show a live signal even under high cytotoxic stress (5 μg mL^−1^ nisin) are also of interest, and the mechanism or strategy by which these cells survive needs to be clarified. Furthermore, single cells whose signal lies between the dead and alive ranges described above can be found at some concentrations, for instance, at 2.5 μg mL^−1^ nisin (Figure 3). It is unclear if the determined signal is constant or if these cells show an intermediate signal because they cannot maintain pH equilibrium and are transitioning from an “alive” to a “dead” state.

A closer observation of the distributions in Figure 3 reveals an increasing number of “dead” or disrupted cells with increasing nisin concentrations. The proportion of disrupted cells was less than 4% when the samples were treated with 0 μg mL^−1^ and 0.3125 μg mL^−1^ nisin and then increased continuously to approximately 10% at 0.625 μg mL^−1^ nisin, 31% at 1.25 μg mL^−1^ nisin and 77% at 2.5 μg mL^−1^ nisin. At 5 μg mL^−1^ nisin, the proportion of disrupted cells reached 98%. Therefore, the distribution can be regarded as concentration dependent, which can also be verified by considering the shown mean value of all measured cells. However, the mean value is particularly interesting for nisin concentrations of 1.25 μg mL^−1^ and 2.5 μg mL^−1^, as the mean is located between the values of the two visible subpopulations and therefore is not suitable for representing the single-cell value of most cells. This does not eliminate the possibility of using this method as an assay for concentration or efficacy determination but does shows that it is based on the signal heterogeneity of the cells. Thus, cell heterogeneity can even be regarded as an underlying mechanism with an important role in the performance of bulk-scale assays using statistical principles of high numbers. This also implies that single-cell and few-cell assays are not feasible because of the lack of significance obtained through heterogeneity. Furthermore, the presence of cells with an intermediate signal should be investigated to determine whether this signal is constant or transient in nature, for example, whether it is derived from cells that were in transition from alive to dead at the time of measurement.

The duration of possible transition signals allows conclusions to be drawn about the effectiveness of the bacteriocin and about the ability of the cells to maintain pH equilibrium despite the presence of pores in the cell wall. Therefore, a time-resolved investigation of signal development using multipoint measurements in microtiter plates and flow cytometry is necessary to clarify outstanding questions and to elucidate the signal development and the dynamics of signal heterogeneity.

### 3.2 Temporal dynamics of ratiometric sensor signal

#### 3.2.1 Microtiter plate-based dynamics on population level

In a subsequent experiment, the ratiometric fluorescence signal of the *L. innocua* biosensor strain after nisin exposure was measured at high temporal resolution in microtiter plates.

**FIGURE 4:**
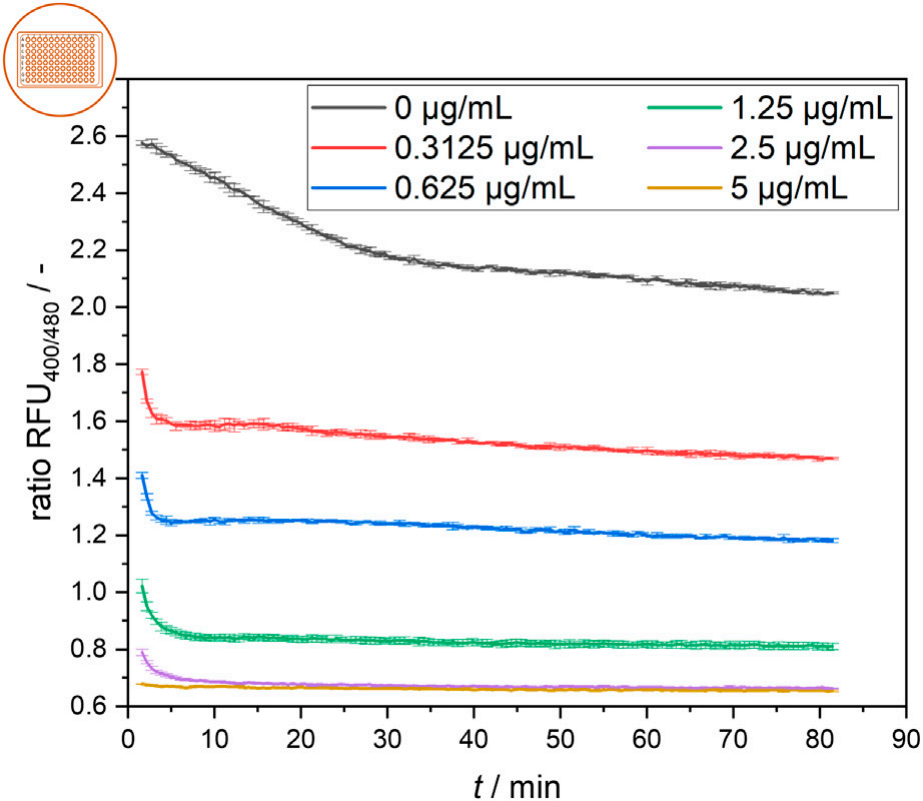
Temporal development of the ratiometric fluorescence signal of *L. innocua* in a microtiter plate setup at different nisin concentrations with respective standard deviations of triplicate measurements.

The signal development of cells treated with different nisin concentrations over time is shown in Figure 4. Generally, the rapid effect of nisin on cells was observed via the distinct decrease in the ratiometric fluorescence signal in all treated samples in the first 5 min after nisin exposure. Cells were affected more quickly by increasing concentrations of nisin, and cells exposed to higher nisin concentrations seemed to reach a constant ratiometric fluorescence signal earlier. At 2.5 μg mL^−1^ nisin, a very rapid decrease in the fluorescence ratio was observed within the first 5 min. Cells in wells containing 5 μg mL^−1^ nisin were almost immediately perforated and maintained at a constant low fluorescence ratio of approximately 0.65. After approximately 30 min, the ratiometric fluorescence signals of all the samples were nearly constant, with the negative control of 0 μg mL^−1^ nisin exhibiting a ratio RFU of approximately 2.1–2.2. The samples were prepared from the same preculture and showed significantly different fluorescence ratios at the first measurement, at 1.5 min, which also demonstrated the rapid effect of nisin on the cells in the first minutes of the assay. Overall, once reached, the fluorescence ratio for each sample remained relatively constant over a 1-hour period. This signal equilibrium suggests that the cells remained in a steady state after approximately 30 min and were not continuously disrupted over time. This result shows that estimating the necessary incubation time for the assay to obtain robust data for concentration determination is essential when designing and conceptualizing novel measurement devices such as online, atline and offline sensor systems or point-of-care tests.

Generally, measurements at the population scale are very reproducible, and the determined fluorescence signals do not vary greatly, which is why heterogeneity within the samples is not noticeable at first glance and is not focused on. Finally, these findings are relevant as the foundation for using the mean flow cytometry signal to determine whether two subpopulations or one homogeneous population with an intermediate signal is present.

#### 3.2.2 Flow cytometry-based dynamics on population level

**FIGURE 5:**
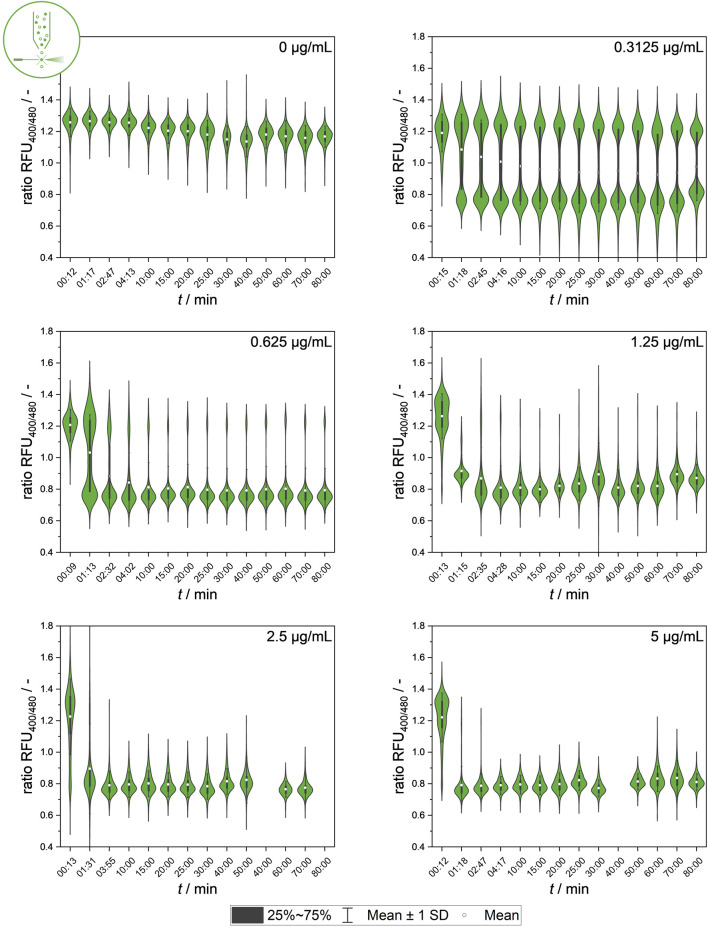
Temporal dynamics of mean values of ratiometric single-cell fluorescence values after exposure to different nisin concentrations (0–5 μg mL^−1^), measured using flow cytometry. Standard deviation of all cells is included as indicator of cell-to-cell heterogeneity.

In the next step, population heterogeneity was analyzed using flow cytometry by quantifying single-cell signal values after different nisin exposure durations to gain further insights into the population dynamics of the development of heterogeneity. Figure 5 shows the changes in population composition. The dataset provides information on the dynamics of the subpopulations and the development of two distinct cell states that cause intermediate ratiometric fluorescence signals in MTP determination at lower nisin concentrations. Additionally, time courses of the mean ratio RFUs with standard deviations as well as the percentage of intact and disrupted cells over time for each concentration are shown in the Supplementary Material in Supplementary Figures S2 and S3.

The temporal dynamics of signal development in single cells at the population level showed clear correlations between the nisin concentration and antimicrobial efficacy against the *L. innocua* strain used. A high nisin concentration, for instance, 5 μg mL^−1^, has an almost instantaneous effect on the population fluorescence signal. The data also show that even at low nisin concentrations, a constant signal is reached after approximately 10–20 min, suggesting that the standard incubation time of 30 min used for bulk-scale measurements could be drastically reduced by up to 66%. This provides insights into the signal development of populations at the single-cell scale, enabling conclusions about whether intermediate signals are uniform signals or mean values produced by heterogenetic behavior. In particular, intermediate fluorescence signals, which mainly occur at intermediate concentrations, are accompanied by higher standard deviations of the fluorescence mean signal, which is caused by heterogeneous single-cell values.

The cause and origin of intermediate signals (ratio RFU values of 0.9–1.2) from single cells need to be further studied. Additional insights into the temporal dynamics and characteristics of single-cell signal development and kinetics will be further researched using a microfluidic setup combined with live-cell imaging.

#### 3.2.3 Microfluidics-based dynamics at single-cell level

**FIGURE 6:**
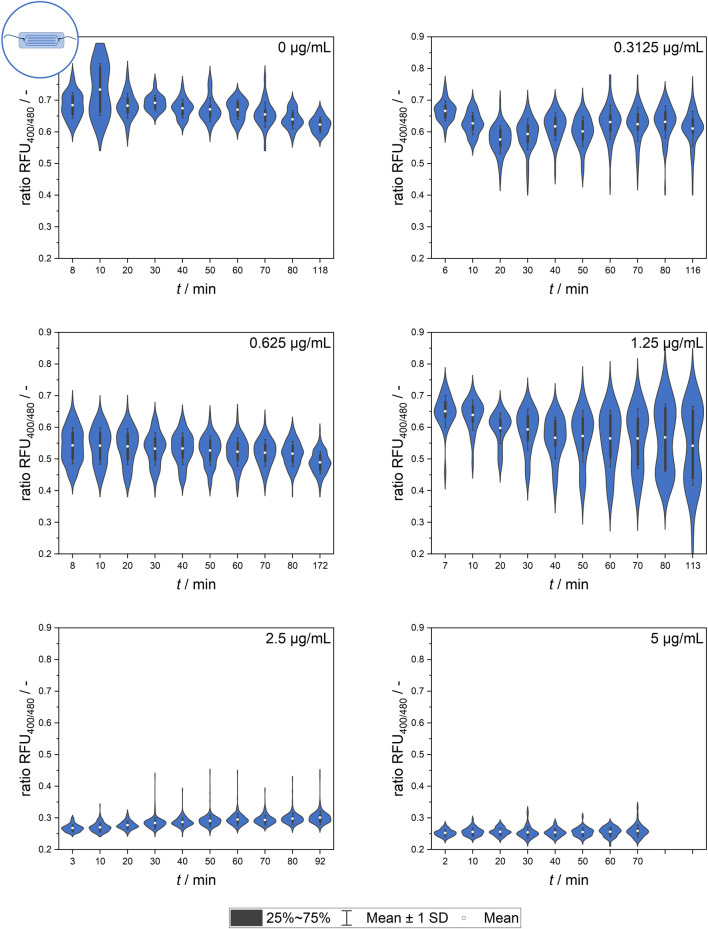
Temporal dynamics of the mean ratiometric single-cell fluorescence values after exposure to different nisin concentrations (0–5 μg mL^−1^) measured using the microfluidic setup. The standard deviation of all cells is included as an indicator of cell-to-cell heterogeneity.

Further experiments analyzing the population dynamics of heterogeneity development by the quantification of single-cell signal values were conducted using the microfluidic setup. Figure 6 shows the mean values of all cells, which are representative of the overall effect of different nisin concentrations on the respective populations.

The signal observed in the control group (0 μg mL^−1^ nisin) remained constant over time at a ratio RFU of approximately 0.7, which is a value corresponding to live, unaffected cells. The slight decrease in the ratio RFU over time might be an artifact of photobleaching effects or biologically induced adjustments of the pH equilibrium as well as environment-dependent sensor sensitivity.

The greatest cell-to-cell heterogeneity was observed at low and intermediate nisin concentrations (0–1.25 μg mL^−1^), and the highest standard deviation occurred at 1.25 μg mL^−1^ nisin. Generally, a time-dependent change in the cell mean signal and heterogeneity was observed for some concentrations. Although the mean signal did not seem to change much over time, the measurement time did appear to affect the analysis. This effect applied especially to the lower and intermediate concentrations, as expected. The data at 1.25 μg mL^−1^ nisin are especially interesting because they suggest the formation of a smaller, nisin-susceptible subpopulation that increases over time.

After the addition of nisin at concentrations above 2.5 μg mL^−1^, the ratio RFU of single cells decreased rapidly. Ratiometric fluorescence values of approximately 0.25, which corresponded to dead cells with a disrupted membrane, appeared a short time after nisin exposure and were already measurable at the beginning of the measurement series, which was consistent with the findings obtained using flow cytometry.

Overall, the datasets from the microfluidic setup and flow cytometer (see Figure 6) revealed similar findings, although the cells showed lower susceptibility to nisin exposure in the microfluidic setups when comparing the ratio RFU as a function of viability. This might have been caused by heterogeneous populations, different nisin batches or slight deviations in standard conditions during preculture cultivation. Another possible reason is the surface contact of the cells in the microfluidic setup, which decreases the free surface area susceptible to pore formation and impaired pH homeostasis. This effect might also occur for targeted cells in real-life applications in which surface contact or biofilm formation might have a large impact on bacteriocin efficacy.

Further investigation of time-dependent heterogeneity, population development and ratiometric single-cell fluorescence values will be necessary to reveal single-cell dynamics.

### 3.3 Temporal dynamics of single-cell fluorescence signals

In the next step, microfluidic experiments were performed to determine the temporal dynamics of single cells. As observed in the results presented thus far, at concentrations of 0 and 5 μg mL^−1^ nisin, cells behaved quite uniformly, and individual single-cell values were nearly indistinguishable from one another (Supplementary Figure S4).

**FIGURE 7:**
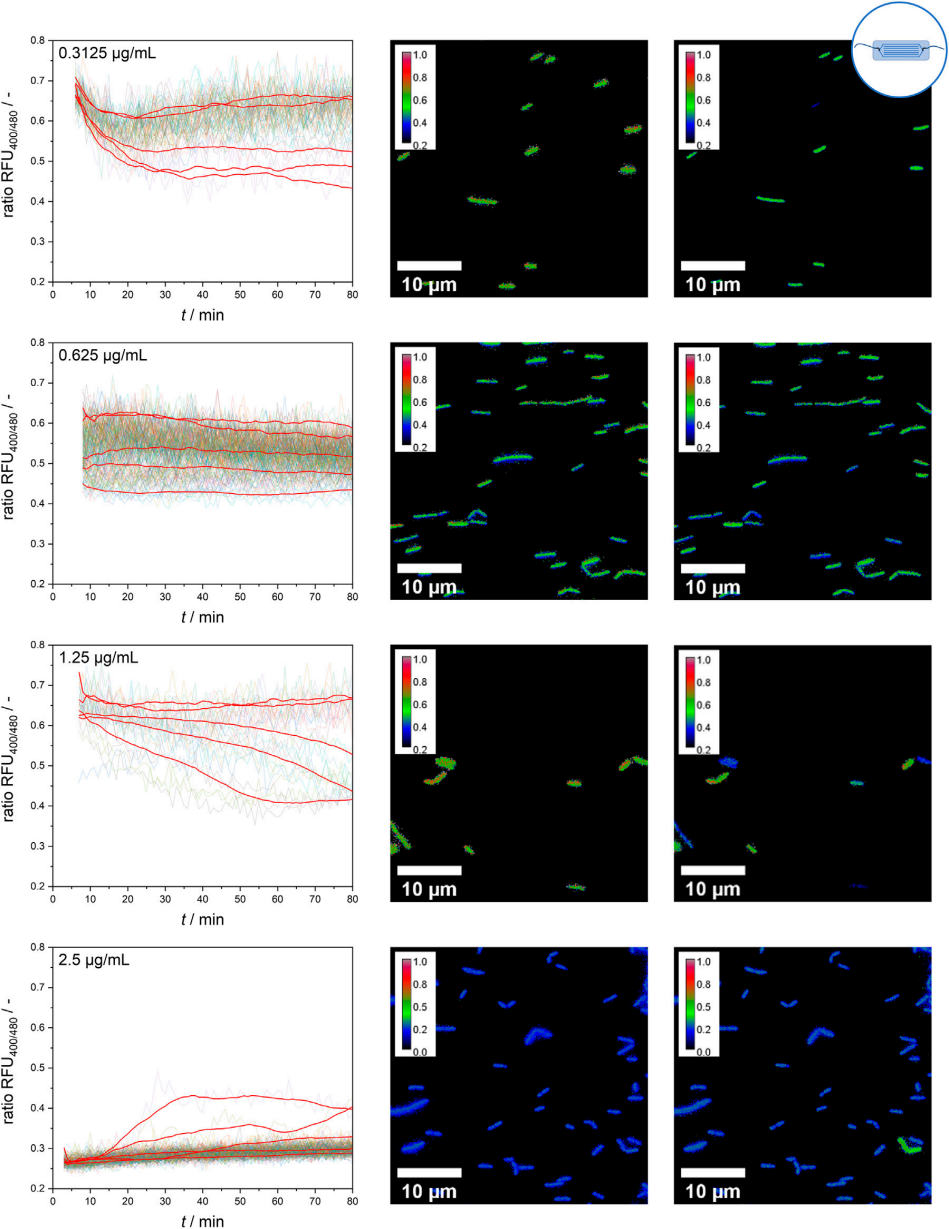
Single-cell signal timelines for intermediate nisin concentrations and ratiometric images at the start and after 70 min of nisin exposure. For each concentration, example signal courses of five different single cells are smoothed and shown in red.

However, at intermediate nisin concentrations (0.3125–1.25 μg mL^−1^), considerable single-cell variability in the temporal response of the sensor bacteria was observed (Figure 7). At a nisin concentration of 0.3125 μg mL^−1^, the population still behaved quite uniformly, but individual cells were susceptible to nisin, causing a decrease in the ratio RFU over time. These cells were outliers, as the majority of cells were only slightly affected by low nisin concentrations. With increasing concentrations, an increasing proportion of susceptible cells and a greater impact of time on cell-to-cell heterogeneity were observed. At a nisin concentration of 0.625 μg mL^−1^, individual cells showed an initial effect after nisin exposure, but the ratio RFU of each cell, and therefore the cell-to-cell heterogeneity, remained almost constant. In contrast, at a nisin concentration of 1.25 μg mL^−1,^ the population showed increasing cell-to-cell heterogeneity over time. This increase was due to the disruption of single cells over time, which increased the proportion of dead cells at subsequent time points. When viewing the temporal course of individual cells, different transition times and dynamics from intact to disturbed pH homeostasis can be observed. Changes in cell-to-cell heterogeneity over time can easily be observed as signal changes of single cells in ratiometric images (Figure 7). Earlier studies investigated *E. coli* after exposure to the antimicrobial peptides LL-37 and Cecoprin A with high spatio-temporal resolved analysis (Sochacki et al., 2011; Rangarajan et al., 2013). The studies revealed concentration and time dependent cell-to-cell heterogeneity as well as heterogeneity in degree of binding and spatial distribution of LL-37 (Sochacki et al., 2011; Choi et al., 2016). Similar underlying effects that caused the heterogeneity in the time lag between peptide addition and permeabilization of the outer membrane in *E. coli* (Choi et al., 2016), could cause changes in cell-to-cell heterogeneity of the *L. innocua* biosensor over time.

An especially interesting finding was obtained at a nisin concentration of 2.5 μg mL^−1^. All cells showed a direct effect and susceptibility to nisin, as expected, which resulted in a ratio RFU of approximately 0.25–0.3 from the start of the measurement. Overall, the majority of cells behaved quite homogenously and shared a similar ratio RFU throughout the measurement time. However, the temporally resolved single-cell data revealed persistent cells that appeared in rare instances and partially reestablished pH homeostasis (verification in ratiometric pictures–Figure 7). The possibility of reestablishing pH homeostasis, which is suggested by the data, indicates the adaptation processes and strategies of the whole-cell biosensor strain. Thus, the ratiometric fluorescence signal of single cells should be carefully analyzed and critically interpreted because even cells with disrupted cell membranes and an unusually low intracellular pH might show persistent behavior in response to their cytotoxic environment and regain pH homeostasis. Nisin-resistant strains of *L. innocua* were reported a long time ago (Maisnier-Patin and Richard, 1996). Abnormal cell wall synthesis and thickened cell walls were assumed to be resistance mechanisms that enabled the resistant strains to withstand high nisin concentrations. In addition, many other mechanisms of nisin resistance have been described for both *L. monocytogenes* and *L. innocua* and might explain these cells that adapted to nisin treatment (Kaur et al., 2011; Bergholz et al., 2013; Zhou et al., 2014; Pang et al., 2022). However, because multiple strategies to cope with nisin treatment have been identified for *L. monocytogenes*, including resistance and tolerance (Zhou et al., 2020) as well as persister cell formation (Wu et al., 2017), further experiments are needed to distinguish the underlying principles and mechanisms involved. More studies investigated persistence and resistance of pathogenic bacteria after treatment with antimicrobial compounds. Differences in the survival rate of single cells and frequency of persister cells might be growth phase dependent. This was observed for *Pseudomonas syringae* pv. *phaseolicola* 1448A after streptomycin and tailocin treatment at different growth phases, i.e., log, early stationary and late stationary phase (Patel et al., 2021). Monitoring of intracellular pH of *Mycobacterium avium* subsp. *paratuberculosis* after exposure to nisin and neutralized cell-free supernatants of *Lactobacillus plantarum* PCA 236 over a time span of 24 h revealed the formation of a probably resistant subpopulation (Gaggìa et al., 2010). Single cell analysis of antibiotic-resistant isolates of *Salmonella enterica* revealed heterogenous gene expression levels and efflux pump activity between cells, suggesting that functional activity and gene expression fluctuations can contribute to adaptive resistances (Sánchez-Romero et al., 2014).

Overall, the data revealed two important findings. First, the mean signal value of the assay arises from the formation of subpopulations and cells with bistable behavior between the signal ratios of intact and disrupted cells. Second, individual persistent or resistant cells were observed that might adapt to nisin-containing environments and regain pH homeostasis. Possible adaptation processes must be investigated and characterized in further experiments.

The methods presented are applicable for characterization and investigation of other bacteriocins and antimicrobial compounds with the same or similar modes of action, for instance pore forming, membrane disrupting, and pH homeostasis influencing compounds. This has already been showed for bacteriocins from class IIa, i.e., Pediocin PA-1 (Chikindas et al., 1993; Khorshidian et al., 2021; Reich et al., 2022) and class IId, i.e., Garvicin Q (Tosukhowong et al., 2012; Desiderato et al., 2022). However, bacteriocins with different modes of action (Perez et al., 2018; Pérez-Ramos et al., 2021; Darbandi et al., 2022), such as DNA replication or protein synthesis inhibiting microcins (Yang et al., 2014), as well as bacteriocins originating from the species of the model organism itself (Darji et al., 1995; Lee, 2020; Meza-Torres et al., 2021) cannot be characterized using the presented biosensor strain. Modified or novel fluorescence-based biosensor strains might be used in combination with the presented methodology for analogous characterization of other antimicrobial compounds and their effects on the respective model organism.

## 4 Conclusion

In addition to flow cytometry, microfluidic single-cell analysis provided deeper insights into the dynamic population and single-cell behavior of the whole-cell biosensor *L. innocua* LMG2785/pNZpHin2^*Lm*^ after nisin exposure. The single-cell data enabled new findings and increased understanding regarding the development and dynamics of cell-to-cell heterogeneity over time.

Cell-to-cell heterogeneity analysis revealed the effectiveness and efficiency of nisin at different concentrations. This may be highly useful for determining the most efficient use of nisin in future applications. Furthermore, the presented methodology can be used to evaluate and characterize novel bacteriocins and their effects on their target organisms as well as to investigate adaptation strategies and persister cell formation.

In combination with flow cytometry, microfluidics provides further insights into the distribution and development of cell-to-cell heterogeneity of the biosensor strain and a better estimation of the influence of heterogeneity on the results of the state-of-the-art MTP assays. The findings could enable an intensive evaluation of assay results and provide evidence for the best measurement time points when using MTP assay methods for quantification. Furthermore, the findings indicate that reproducible nisin concentration or efficacy assays at the single-cell level are intrinsically difficult due to the formation of subpopulations and the possibility of adaptation strategies for individual cells. This is particularly relevant for assays with small numbers of cells in single-cell analysis platforms, such as microfluidic setups. Therefore, bacteriocin efficacy and concentration assays can be conducted at the single-cell level only when a high-throughput measurement method, such as FC, is chosen to average the effects of heterogeneity at the single-cell level with an appropriate sample size.

## Data Availability

The raw data supporting the conclusion of this article will be made available by the authors, without undue reservation.
