# Supplementary material for: Time-resolved cell-to-cell heterogeneity of Listeria innocua after nisin exposure
Source: Front Bioeng Biotechnol. 2024 Jun 12;12:1408652. doi: 10.3389/fbioe.2024.1408652 (PMC11199691; doi:10.3389/fbioe.2024.1408652)
Supplement: Supplementary file 1 [file DataSheet1.pdf]

Supplementary Material

Time-Resolved Cell-to-Cell Heterogeneity of *Listeria innocua* after Nisin Exposure

Niklas Fante[1], Christian K. Desiderato[2], Christian U. Riedel[2] and Alexander Grünberger[1,3,4*]

[1] Multiscale Bioengineering, Technical Faculty, Bielefeld University, Bielefeld, Germany

[2] Department of Biology, University of Ulm, Ulm, Germany

[3] Center for Biotechnology (CeBiTec), Bielefeld University, Bielefeld, Germany

[4] Institute of Process Engineering in Life Sciences: Microsystems in Bioprocess Engineering, Karlsruhe Institute of Technology, Karlsruhe, Germany

*** Correspondence:** alexander.gruenberger@kit.edu (A. Grünberger)

1 Supplementary Figures

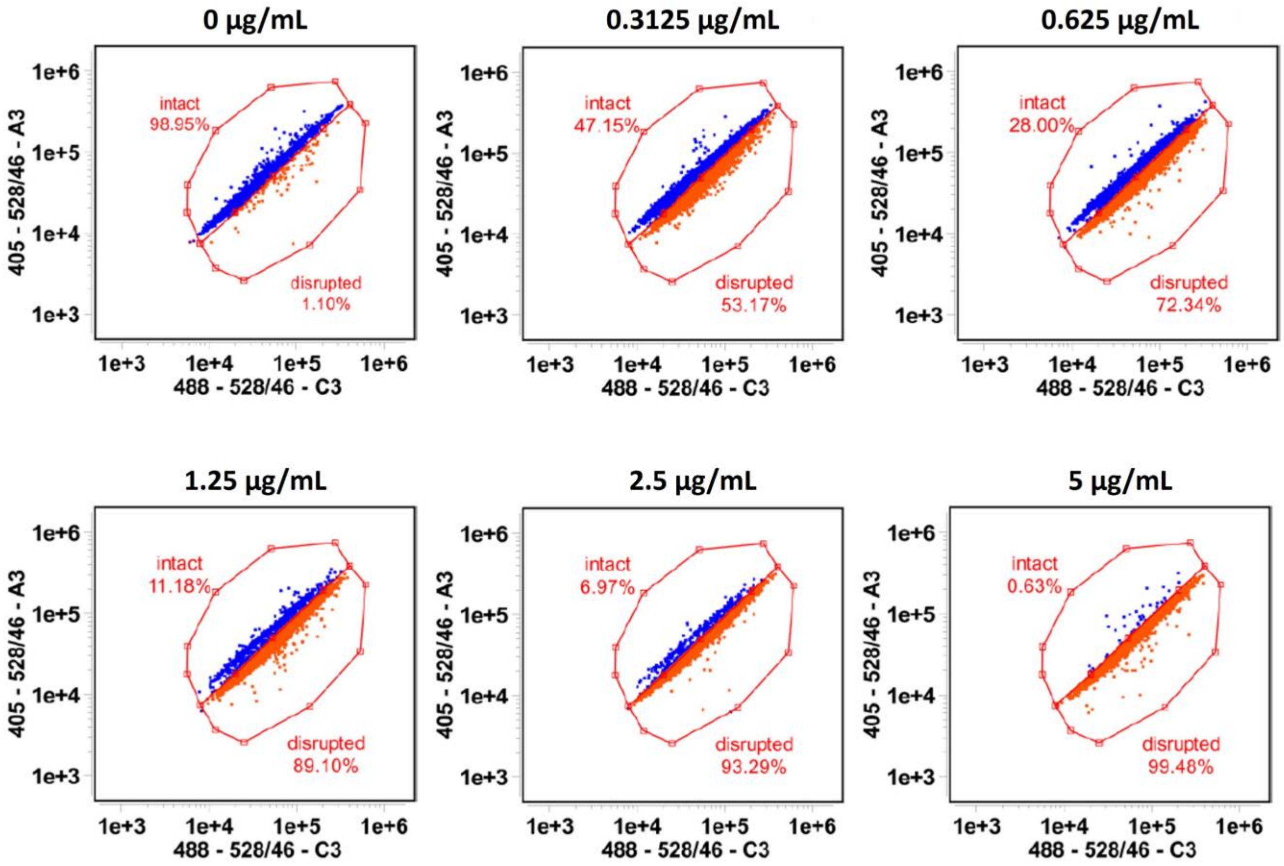

Figure S1. Gating and results of flow cytometry-based single-cell analysis. Gating of single-cell ratio RFU from flow cytometry measurements for samples treated with different nisin concentrations (0–5 µg mL^{-1}) for 30 min.

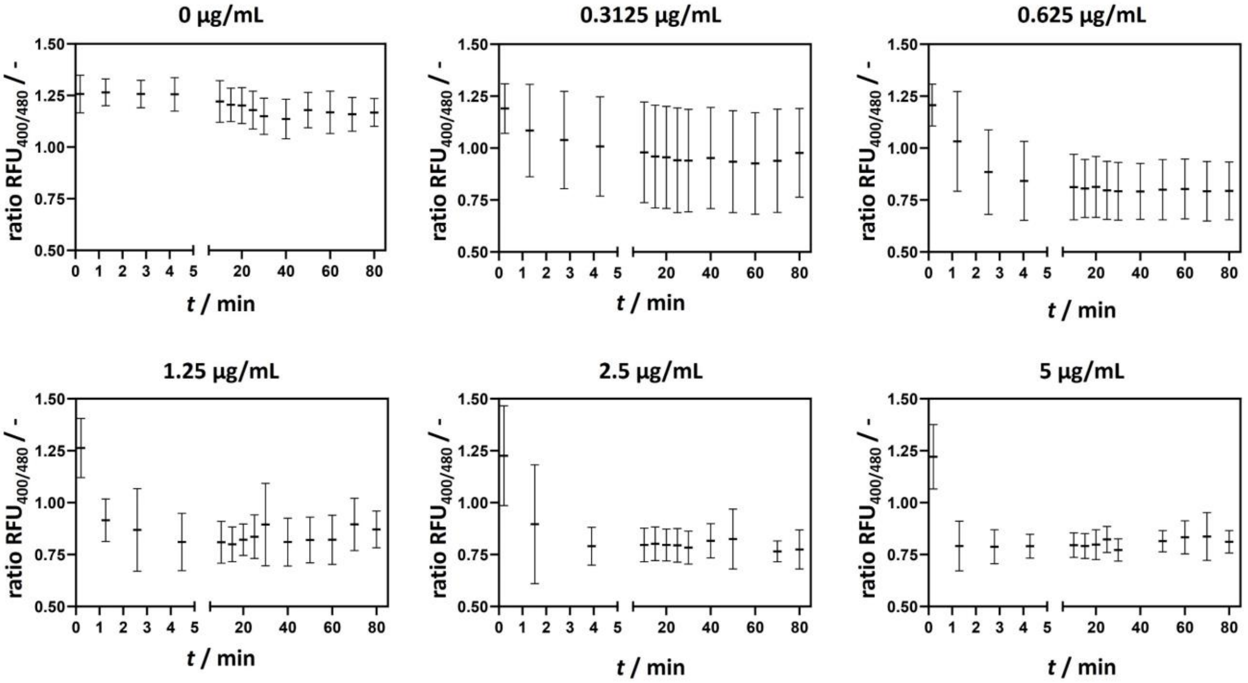

Figure S2. Time-resolved results of flow cytometry-based dynamics on population level. Time course of the mean ratio RFU and standard deviation of all cells after exposure to different nisin concentrations (0–5 µg mL^{-1}) determined using FC.

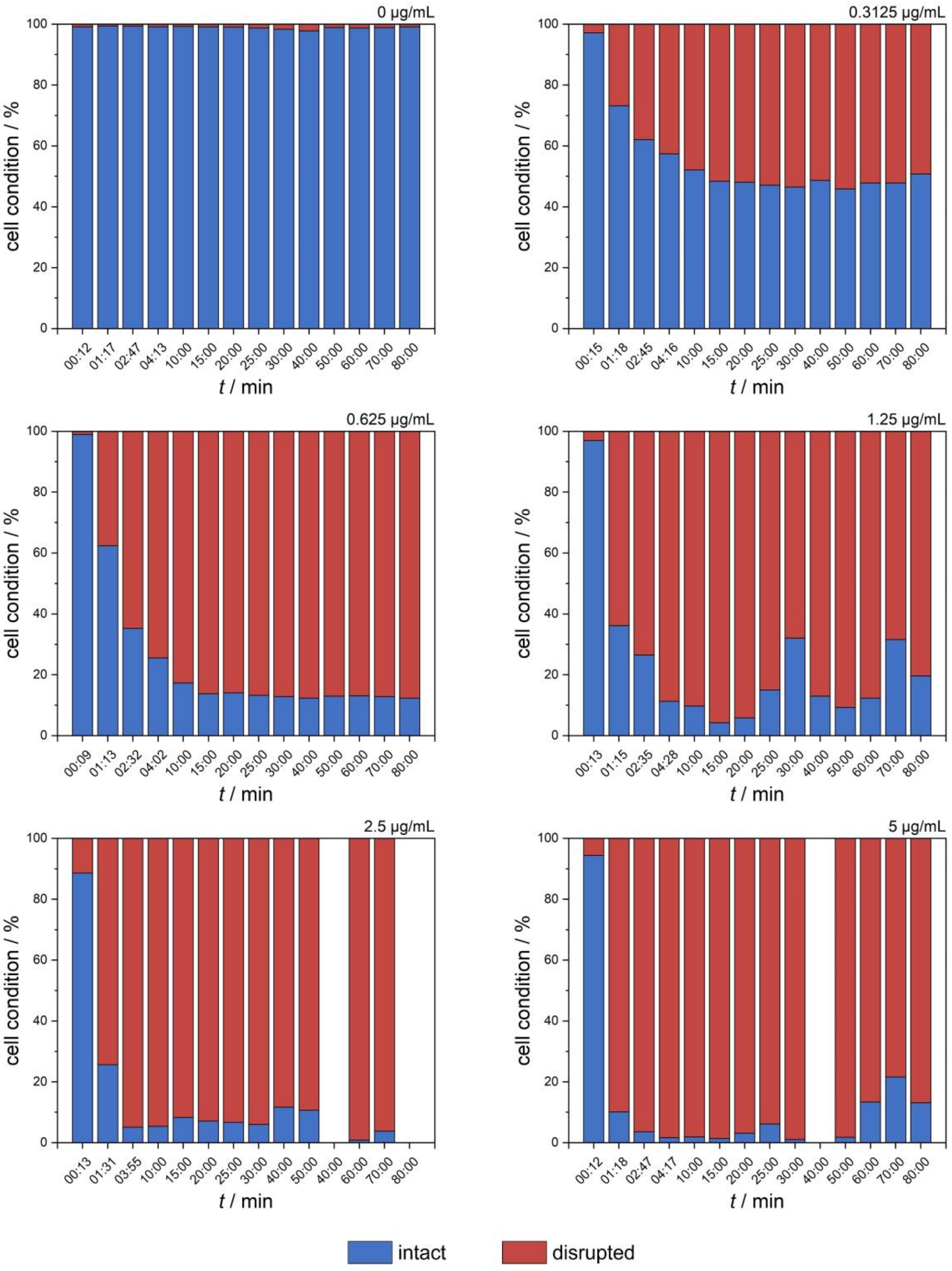

Figure S3. Time-resolved results of flow cytometry-based dynamics on population level after subpopulation classification. Time course of the percentage of intact and disrupted cells of *L. innocua* after exposure to different nisin concentrations (0–5 µg mL^{-1}). Classification of cells is based on gating results of flow cytometry data.

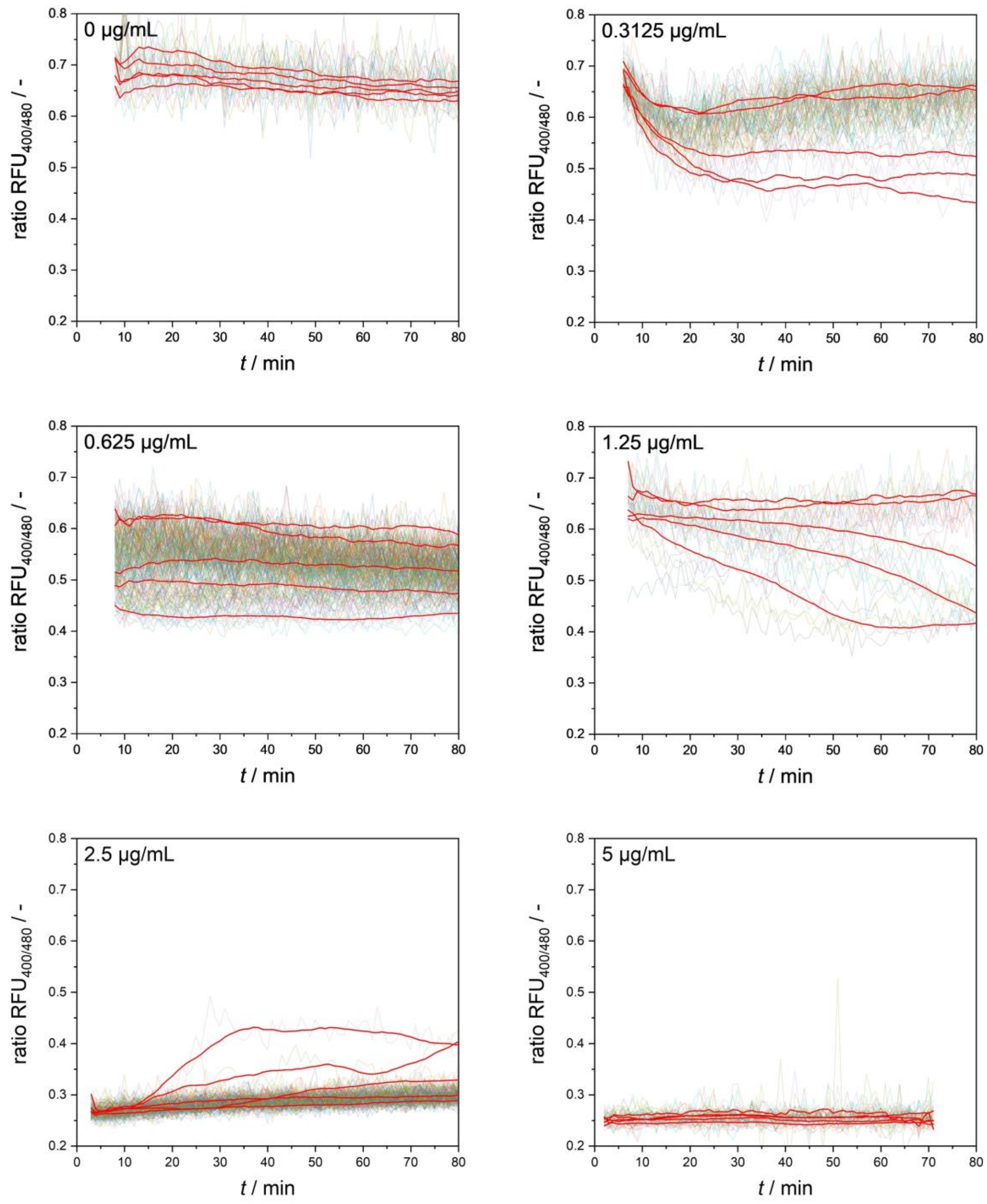

Figure S4. Microfluidic live-cell imaging experiments give insights into temporal dynamics of single-cell fluorescence signals. Time course of the ratio RFU of single cells after exposure to different nisin concentrations (0–5 µg mL^{-1}). Exemplary signal courses of five different single cells are smoothed and shown in red for each concentration.
